# Traditional and Artificial Intelligent Methods in Predicting Maxillofacial Soft Tissue Morphology After Orthognathic Surgery: A Narrative Review

**DOI:** 10.1155/ijod/6268492

**Published:** 2025-10-30

**Authors:** Tianyi Wang, Huanhuan Chen, Guangying Song, Bing Han

**Affiliations:** Department of Orthodontics, Cranial-Facial Growth and Development Center, Peking University School and Hospital of Stomatology, 22 Zhongguancun South Avenue, Haidian District, Beijing 100081, China

**Keywords:** artificial intelligence, deep learning, machine learning, maxillofacial soft tissue morphology, orthognathic surgery, orthognathic surgery planning, postoperative soft tissues

## Abstract

The soft tissue morphology after orthognathic surgery has always been difficult to predict in preoperative planning. This narrative review aims to evaluate the current prediction methods and point out the direction for future research to improve predicting accuracy and speed. PubMed was the database for the review. The keywords for searching include: orthognathic surgery, prediction methods, simulation methods, artificial intelligence (AI), machine learning (ML), deep learning (DL), soft tissue, and surgical planning. Sixty-seven articles were found and 60 relevant articles were evaluated. The review finds out that with the transition from two-dimensional (2D) prediction to three-dimensional (3D) prediction, AI algorithms, especially DL methods, are brought forward promisingly, such as deep neural network (DNN), PointNet, and Transformer. DL methods have the advantages of high accuracy, fast speed, and simplified use, therefore have broad development prospects. However, DL methods need to be further improved, mainly in data insufficiency, overfitting, and the lack of interpretability. To conclude, the review proposes methods that could improve the accuracy and availability of prediction by DL, such as learning from large and high-quality databases, establishing separate databases for groups with different features, optimizing and simplifying the algorithm structure, utilizing integrated multimodal data, and implementing visualization techniques.

## 1. Introduction

In the past three decades, orthognathic surgery combined with orthodontics has been widely used to treat severe dentofacial deformities [1]. Generally, preoperative planning is the most important step in the workflow of orthognathic surgery [2]. The soft tissue morphological changes after orthognathic surgery have always been difficult in preoperative prediction. Accurate prediction of soft tissue is important for preoperative patient counseling, managing patient expectations, improving outcomes, providing reference for surgeons in goal-driven planning, and planning the second stage of soft tissue surgery [3].

However, the prediction of soft tissue appearance after orthognathic surgery is difficult to be accurate [4]. The complex morphology and internal structure of facial tissues, the 3D bone movement, the sophisticated involvement between soft and hard tissues make them difficult to predict. The lip region is closely related to the patient's esthetic perception and satisfaction with surgery, but it is also the most difficult part to predict. Due to different lip length, tonicity, posture, and mass [5, 6], the accuracy of current prediction methods is often beyond the acceptable range [7]. Accurate prediction of the appearance of this subregion is a key point in the development of prediction methods [8].

Traditional surgical planning (TSP) provides prediction of 2D postoperative soft tissue morphology. Generally, it is based on the amount of movement of the corresponding bone and calculated by the ratio of change of soft tissue to hard tissue [6, 9]. TSP in orthognathic surgery has limitations due to the use of 2D X-rays and dental models, which is time-consuming and often results in cumulative errors [10]. With the development of 3D technology, virtual surgical planning (VSP), which can perform 3D prediction, is rapidly rising [11]. VSP is based on a 3D virtual model that simulates the actual maxillofacial bone and dental arch structure of the patient [12]. Studies have shown that using computer-aided design (CAD), computer-aided manufacturing (CAM) technologies, and advanced imaging techniques, VSP can create precise orthognathic plans, reduce human errors, improve surgical precision, and improve patient satisfaction and recovery [11]. Traditional 3D predicting methods, such as the ratio of soft tissue to hard tissue changes, regression equation, and biomechanical simulation, have the problems of low prediction accuracy, slow speed, and high cost, which are difficult to meet the clinical needs, and their implementation is still mainly limited to specialized hospitals [13]. Moreover, the requirement of trained technicians [14], the small size of 3D databases, the limitations of software functions, and the lack of in-depth understanding of 3D data analysis are still major obstacles to its widespread use.

AI generally refers to the ability of machines to mimic human cognition and behavior. In recent years, it has developed rapidly in the field of medicine in terms of complexity, diversity, and computing power and has become one of the most promising technology research and development directions [15, 16]. AI made research progress in predicting the soft tissues after orthognathic surgery mainly by machine learning (ML) [17]. ML and deep learning (DL) are implementations of algorithms that analyze data and induce models describing certain properties of this data, allowing for future predictions on new sets of data. Thus, ML and DL can be defined as computational systems that learn over time based on experience. Prediction methods based on deep neural network (DNN), PointNet, Transformer, and other principles have emerged, but are still in their infancy.

The most representative existing review in post-orthognathic surgery soft tissue predicting [8] starts from different prediction software, and the prediction algorithms mentioned in the available reviews [8, 18] are mostly traditional methods without AI. Based on the comparison of different algorithms, the present review makes a comparative analysis from the fundamental algorithm principle level, which makes it more conducive to find out the appropriate method and achieve the ultimate goal of improving the prediction effect. In addition, this review summarizes the latest research progress of AI in predicting postoperative soft tissue in recent years, compares and analyzes the most cutting-edge research results, and tries to find out the future research direction to further improve the effect of AI prediction.

## 2. Materials and Methods

The manuscript screening process was led by Wang T, who is an experienced researcher with expertise in orthodontics, so as to meticulously evaluate the manuscripts based on predefined criteria to ensure the selection of high-quality and relevant studies for inclusion in this review.

This narrative review was conducted in accordance with a predefined protocol that outlined the objectives, methodology, and criteria for study inclusion and exclusion. The protocol was developed based on the guidelines for conducting narrative reviews and was designed to ensure the rigor and transparency of the review process. The main objectives of the review were clearly stated, focusing on the prediction of soft tissue morphology after orthognathic surgery. The inclusion criteria specified the types of studies to be included, such as reviews, randomized controlled trials, and observational studies. The exclusion criteria were: (1) articles with the sample of cleft lip and palate, (2) articles with the sample of nasal deformities, (3) case report, and (4) studies published in languages other than English.

To ensure a comprehensive search, a set of carefully selected keywords was used in combination with relevant medical subject headings (MeSH) terms. The primary keywords included orthognathic surgery, prediction methods, simulation methods, artificial intelligence (AI), ML, DL, soft tissue, surgical planning, and they were combined using Boolean operators (AND, OR) to broaden or narrow the search scope as needed.

The selection process of the manuscripts was conducted in a rigorous manner. Initially, a comprehensive search was performed in the PubMed database, to identify potential studies. Sixty-seven articles were found. The search results were then exported to a reference management software for further processing. The first step involved the removal of any duplicate records to avoid redundancy. Subsequently, the titles and abstracts of the remaining studies were screened by the operator to assess their relevance to the review topic. Seven studies that did not meet the inclusion criteria were excluded at this stage. For the remaining studies, full-text articles were retrieved and thoroughly reviewed to determine their final eligibility. For uncertainties during the screening process, Chen H and Song G were consulted to make the final decision.

Once the eligible studies were identified, the data extraction process was initiated. A data extraction form was developed to collect relevant information from each study, including study characteristics (e.g., authors, publication year, study design), population details (e.g., sample size, demographics), predicting method and accuracy. The extracted data were then aggregated and synthesized in a narrative manner. This involved a thorough examination of the findings from the included studies and identifying common predicting methods, patterns, and overall trends. The data were organized into different sections based on the prediction method. A detailed narrative summary was then provided for each section, highlighting the key algorithm, strengths, and limitations of the studies. The narrative review also included a discussion of any potential improvements for these predicting methods. Additionally, the implications of the findings for clinical practice and future research were also discussed based on the synthesized data.

## 3. Results

**Figure 1:**
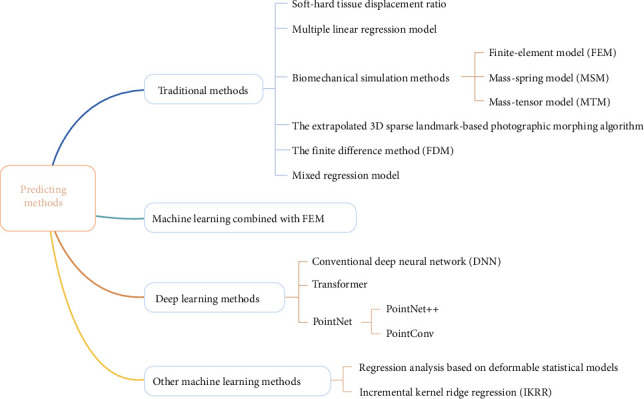
Classification of current methods in predicting maxillofacial soft tissue morphology after orthognathic surgery.

The classification of current methods in predicting maxillofacial soft tissue morphology after orthognathic surgery is shown in Figure 1.

### 3.1. The Trend and Necessity of the Implementation of AI

Nowadays, 2D prediction algorithms are the most widely used prediction methods in clinical practice. However, the 2D predicting accuracy of commonly used software, such as Dolphin Imaging software (Dolphin Imaging & Management Solutions, Chatsworth, CA, USA), is not high, and the lower lip prediction is the least accurate [19]. Besides, 2D soft tissue prediction is not reliable enough when the complexity of surgery increases [8]. Although there is no consensus on the best 3D prediction model, it has been suggested that 3D prediction may be more accurate and have greater clinical value compared to 2D prediction [20].

Accuracy for prediction is commonly calculated by generating predicted soft tissue and, subsequently, comparing the simulation with the actual postoperative soft tissue contour by mean absolute distances [21–24]. In clinical application, linear difference above 2 mm was considered a significant difference and used as a criterion for accuracy [25]. However, even if individual errors are below 2 mm, the composite addition of these errors may be clinically meaningful. Hemelen et al. [26] reported that VSP had higher accuracy than TSP methods. The 3D prediction errors in coronal direction were reported to average 1–1.5 mm [9] with less residual asymmetry compared to TSP [27], so the VSP technique should be used preferentially over the TSP technique [3, 18].

However, the accuracy of 3D prediction is controversial [28, 29]. Olivetti et al. [8] reported that 3D methods are inferior to 2D methods in terms of accuracy and surgeon satisfaction and fail to meet medical needs. The surgeons' opinion was that the prediction was mainly to provide a possible postoperative appearance display. It has been reported that most of the prediction inaccuracy originates from soft tissue uncertainty [6] and increases when the complexity of the procedure increases [8]. In addition, 3D prediction by software programs is limited to highly specialized hospitals for it is time-consuming and requires training [30]. To solve these problems, we need to find the most promising algorithm from the perspective of principle and take improvement measures.

### 3.2. Traditional Methods

The most fundamental idea of soft tissue prediction is based on the displacement of the corresponding bone and is calculated by the displacement ratio of soft tissue to hard tissue [31]. However, it has been shown [4] that there is no precise proportional relationship between soft tissue motion and underlying bone motion.

Multiple regression analysis generally take hard tissue information as the independent variable and soft tissue information as the dependent variable to obtain the prediction equation for soft tissue markers. The average prediction error for horizontal lip position was reported within 1.04–1.51 mm by Lu et al. [32]. The regression model was more accurate than simple proportion prediction, but the variability of individual prediction results was high [33]. This method predicts the change of a limited number of points or measurements without generating contour. However, the number of variables in the equation is usually small, which is convenient to use in practice.

Biomechanical simulation methods are currently the main software-based methods for predicting facial morphology, including finite-element models (FEMs) [9, 34], mass-spring models (MSMs) [22, 28], mass-tensor models (MTMs) [7, 21], etc. MSM and MTM methods discretize objects into masses and springs, with relatively little computational time required [35]; disadvantages include the lack of a biomechanical basis [34]. The spring constants have no direct correlation to material properties. FEM, in which properties can be assigned to each element [34], is more biomechanically relevant and accurate, but it requires longer simulation time and higher calculation cost [36, 37], and the success of FEM simulations depends on a high-quality mesh model of soft tissues [38]. It has been shown that 3D prediction techniques based on FEM are superior to 2D software programs in predicting the lower third of the face, especially the lip and chin regions [39]. The FEM method improved by Kim et al. [36, 37] was more accurate than the traditional FEM method, using FEM with realistic lip sliding effect (FEM-RLSE). Knoops et al. [34] proposed a probabilistic (instead of a deterministic) FEM approach by which the postoperative range of soft tissues was predicted. In addition, some researchers increased the accuracy by considering the details of soft tissue (muscle) modeling [40]. In order to reduce the time-consuming of simulation, Zhang et al. [41] proposed a new semi-automatic method to deform the template and generate patient-specific facial soft tissue models efficiently and accurately, including internal structure (muscle) modeling.

The extrapolated 3D sparse landmark-based photographic morphing algorithm is based on sparse landmarks and is incorporated into Dolphin Imaging software for 3D prediction. It is essentially extrapolated from 2D predictions. Its main feature is that it allows setting patient-specific bone-to-soft tissue ratios. However, studies [29, 42–44] have shown that prediction accuracy of this algorithm for the upper lip, nasal floor, and subalar area is clinically unacceptable.

The finite difference method (FDM) [45], unlike the method based on sparse landmarks, has no manual setting for specific material properties. This method is incorporated in ProPlan CMF software (Dentsply-Sirona, York, PA, USA). Knoops et al. [29] suggested that FDM provided better 3D prediction of continuous displacement.

A mixed regression model was used by Yamashita et al. [46] to predict landmark coordinate in surgeries, and the covariates gender, age, and prediction accuracy were used as predictor variables. They believed that the generalized linear model was defective in principle because the independent variables of the prediction model did not meet the principle of data independence and were more likely to be nested data. The prediction accuracy of the model for Class II patients was lower than that for Class III patients, and the error of some landmarks was more than 2 mm.

### 3.3. ML Methods Combined With FEM

There have been a number of studies that have proposed ML methods to help reduce the time required for FEM to achieve real-time responses on the millisecond scale [47]. The preferred ML method is neural network (NN) in DL, one of the main reasons is that NN has been proven to accurately simulate nonlinear functions of biomechanical behavior of various anatomical structures [48]. However, obtaining adequate datasets for DL algorithms is challenging. Therefore, most of these methods require training by FEM-simulated data [47, 48]. However, FEM has assumptions and simplifications for the real situation, which may lead to inaccurate DL simulation results. Thus, the most ideal approach should still be to let AI directly learn from real patient data [47]. Zhang et al. [49] believed that biomechanical simulation did not consider population-based statistical data, nor did it consider the nonlinear, time dependent, and anisotropic behavior characteristics of living tissues. Therefore, they proposed an integrated biomechanical and statistical learning model. Its average prediction error was lower than FEM [35].

### 3.4. DL Methods

Among ML methods, DL algorithms are more suitable for analyzing large or complex data and are the main method for predicting soft tissues after orthognathic surgery [17, 50]. DL learns patterns in data structures through NNs [51, 52], a set of algorithms that simulate the functional patterns of the human brain to solve problems [53, 54].

The accuracy of the DL model was higher than that of the biomechanical models, and its accuracy has the potential to be further improved in future studies. DNN, Transformer, and PointNet are currently the main three DL architectures in orthognathic soft tissue prediction. Based on NN, they learn from constantly adding new data, so they are expected to improve from gradually increasing samples. They have extensibility and flexibility to suit different task requirements, yet rely on large amounts of data for training. These commonalities embody their core properties as DL models to automatically learn features and patterns in data through NN architectures, so as to achieve effective processing of complex tasks. Inclusion of additional variables (patient-specific factors) in DL models, such as age, sex, and presence of orthodontic appliances, would also help to improve prediction accuracy.

**Figure 2:**
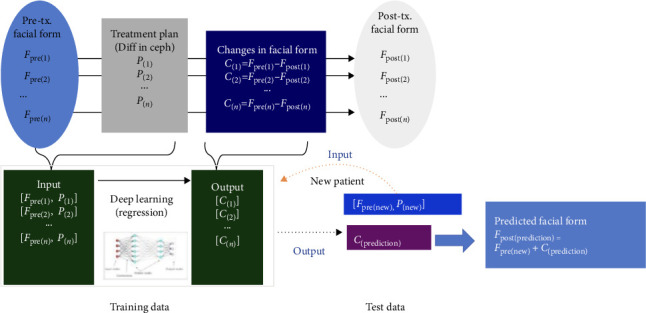
The mathematical model used to develop the AI systems [57].

DNN is an architecture that consists of an input layer, multiple hidden layers, and an output layer [23]. Each layer consists of multiple neurons, which are connected by weights. The core feature of DNN is to extract high-level features of data through multi-layer nonlinear transformation, so as to realize complex function mapping. DNN is generally used in postoperative prediction because of its higher accuracy than traditional method such as MTM [55]. However, the standard deviation of DNN was large, especially for the lower lip and chin [23]. Meanwhile, the DNN training was difficult based on 3D coordinates of thousands of soft tissue nodes and hard tissue displacement [23, 56, 57]. Geometric morphometrics (GMM) uses dense corresponding vertices of skull and face in order to capture more information than manually identified points and has been widely used in facial shape analysis [58]. Tanikawa et al. [57] trained DNN combined with GMM to predict the position of thousands of soft tissue semi-landmarks (Figure 2), with an average system error below 1 mm. Moreover, DNN can be combined with other models (e.g., Transformer) to further improve the performance. However, DNNs have limitations such as difficult training, poor interpretation, and high computational complexity.

**Figure 3:**
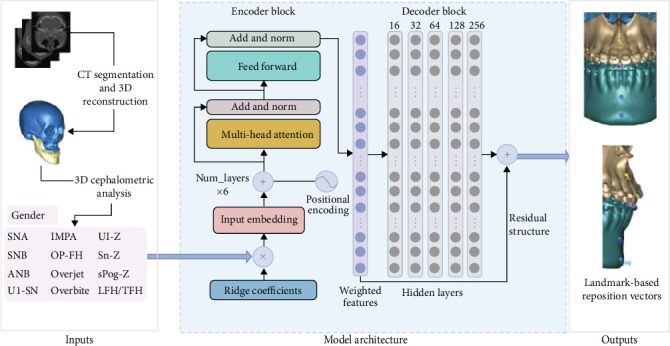
Architecture of VSP Transformer [59]. Norm: normalization. CT-reconstructed model was quantified using 3D cephalometric analysis. 12 cephalometric variables and gender were weighted using ridge coefficients. After embedding, the data were encoded with positional information and then input into encoder block to extract features. At last, the extracted features were decoded by a multi-layer perceptron (MLP) and an embedded residual structure to obtain output variables.

The Transformer NN model is a DL architecture that improves training and inference efficiency through the self-attention mechanism and the advantages of parallel computing. The self-attention mechanism is a core component of Transformer, which allows the model to process each position in the sequence while simultaneously considering information from other positions in the sequence. The architecture of Transformer consists of encoder and decoder, both of which are built based on self-attention mechanism. Cheng et al. [59] developed a Transformer to automatically predict ten coordinate-based displacement vectors of jawbones (Figure 3). The mean absolute errors of prediction were better than classical regression models.

The most common method for processing 3D soft tissue surface structure is to decompose soft tissue surface data into dense point clouds for analysis and prediction. PointNet is a pioneering point cloud processing architecture that directly operates on the point cloud data through point-by-point feature extraction and global feature aggregation, preserving the original information and flexibility of the point cloud [38]. PointNet++ extends PointNet by hierarchically learning spatial features in a more efficient and powerful way. Ma et al. [60] proposed a weakly supervised point-displacement network based on PointNet++ and was trained using pairwise data with strict point-to-point correspondence. They fed bone motion vectors and preoperative facial point cloud to the network to predict facial point cloud motion vector and generate postoperative facial point cloud. The results showed that the accuracy of PointNet++ in predicting facial changes was comparable to that of FEM-RLSE method, and it was significantly faster. Point-wise convolutional network (PointConv) that comprehensively captures the complex geometric correspondence between skeletal and facial shapes was used for dynamic point-wise convolution. Ma et al. [61] trained PointConv [62] to predict facial shape from bony shape and greatly improved the prediction accuracy compared with PointNet++.

### 3.5. Other ML Methods

Deformable statistical model was used to perform regression analysis in soft tissue prediction. The core idea is that shapes such as faces can be obtained by weighted addition or multiplication of many independent features (basis vectors that are orthogonal to each other). The prediction using this method relies only on external preoperative and postoperative facial morphology and does not require CT as input [63]. Knoops et al. [24] proposed the first large-scale clinical 3D morphable models (3DMM), a framework based on supervised learning and achieved satisfying accuracy of fully automated simulated surgical results.

Soft tissue deformation is related to the biomechanical properties of soft tissue and bone. Pan et al. [13] established a incremental kernel ridge regression (IKRR) model to describe the relationship between postoperative soft tissue deformation and biomechanical characteristics in order to incorporate population-based statistics. Ridge regression is an extension of linear regression in which the ridge parameter is used to prevent model overfitting [64]. The average prediction error (0.9103 mm) was lower than some state-of-the-art algorithms.

## 4. Discussion

Comparison between studies evaluating the accuracy of soft tissue prediction is difficult because measurement metrics, type of surgery, and software were different [46]. As for the factors influencing the accuracy of prediction, in addition to prediction methods, there are biological factors [65], age, gender [46, 66], ethnicity [67], database of preoperative soft tissue thickness and average ratio of soft and hard tissue movement changes, amplitude of bone movement, and condylar displacement. Some of these factors are unavoidable, while others can be difficult to control and predict.

The intelligent simulation program of DL can reduce the requirements for personnel training and operation, shorten the predicting time, and increase the cost efficiency. At present, the accuracy of automated DL algorithms is insufficient, especially for severe deformity cases, which makes them difficult to enter clinical application. The major problems in the development of DL algorithms are data insufficiency, overfitting, and the lack of interpretability.

### 4.1. Data Insufficiency

DL models have a large number of parameters and require a large amount of data to learn the optimal values of these parameters, without which the model may not be able to sufficiently learn regularities and patterns in the data. In order to improve DL prediction accuracy, increasing the size of the training sample is crucial [61]. The proper strategies include data augmentation, transfer learning, and semi-supervised training [15, 16]. Data augmentation generates more training samples by applying transformations (such as rotation, scaling, and cropping) to the existing data. Transfer learning leverages a model pre-trained on a large-scale dataset and then fine-tuned on a smaller dataset. Semi-supervised learning combines a small amount of labeled data with a large amount of unlabeled data to train a model. Semi-supervised learning is very effective when labeled data is scarce but unlabeled data is abundant. Pan et al. [13] recommended a semi-supervised method in which the training set consisted of a large amount of preoperative data and a small amount of postoperative data. Ma et al. [61] proposed a two-step sampling strategy that could generate multiple sets of data from one patient for training. Alternatively, unsupervised methods that require smaller sample size have been tested [68], although their use has not been demonstrated in patients. In addition, considering the potential of using smartphones to take 3D scans [69], there is an opportunity to replace expensive facial scanning equipment and promote sample accumulation.

Attention should also be paid to the quality of the sample. For example, patients of different ages, genders, and races should be included. Moreover, the demographic characteristics of the sample, such as male/female 1:1 ratio, can more comprehensively reflect the law of different genders. Besides, real patient data are better than simulated data. The sample should also include sufficient cases of different surgical procedures, severe deformity cases [38], and long-term follow-up data. By substituting 3D facial scan data for the soft tissue surface in cone-beam computed tomography image, integrated multimodal data provides higher accuracy in soft tissue information.

Future improvements in DL prediction algorithms require large and multicenter databases with various surgical types and more diverse surgery set [57, 59]. The suggested approach is to develop cloud-based databases with shared resources globally [70]. On the basis of this global database, the next move is to establish separate databases for groups with different surgical methods, skeletal types, gender, age, and ethnicity, which is beneficial to improve the prediction accuracy of specific patients. In addition, the credibility and practicability of the prediction model should be shown by validation dataset based on real patients [71].

### 4.2. Overfitting

The complexity of facial anatomy often leads to the high complexity of the model [57], so that the prediction is disturbed by the overfitting problem. Overfitting is the phenomenon in which a model performs very well on the training data with low error but performs very poorly on new, unseen data (test data or data in real-world applications), hence, reduces the usefulness of the model in clinical practice [15]. This is usually because the model is so complex that it learns not only the true regularities in the data, but also the noise and outliers in the data. Insufficient amount and low quality of training data can also lead to overfitting [15].

Regularization techniques [72] can effectively limit the complexity of the model and prevent the model parameters from being too large and causing overfitting. Cross-validation [15] splits the training data into *k* subsets, and then perform training and validation *k* times, avoiding the model only performing well on a fixed training set. Simplifying the model structure will reduce the complexity of the model and reduce the risk of overfitting. For NNs, it is possible to reduce the number of layers of the network and the number of neurons in each layer. Early stopping [73] is to stop early during training when the model no longer improves performance on the validation set, can also help to avoid overfitting.

### 4.3. The Lack of Interpretability

In the prediction of facial soft tissue after orthognathic surgery, the interpretability of DL models is crucial, to ensure that the decision-making process of the model is transparent and understandable. The lack of interpretability often causes confusion in clinical applications, making it difficult for doctors to grasp the internal rules and explain the causes or mechanisms of soft tissue changes to patients. Reasons for the lack of interpretability in DL include model complexity, the data-driven nature [15], and the opacity of the training process.

Postoperative changes in soft tissues are mainly caused by intraoperative changes in hard tissues. In the long run, to achieve higher accuracy and interpretability, algorithms for predicting soft tissue changes should seek association mechanisms from preoperative and postoperative soft tissue and hard tissue changes and train models [60], instead of only learning from preoperative and postoperative soft tissue surface data [63]. In this way, integrated multimodel data [74] can help the model better understand the complexity of facial soft tissues.

Visualization techniques are suggested for interpretability improvement. By visualizing the feature maps of the middle layers of a NN, it is possible to intuitively understand how the model is processing the input data. For example, in a convolutional NN (CNN), feature maps of convolutional layers can be shown to help understand how the model extracts facial features step by step [15]. For models that incorporate attention mechanisms such as Transformer [59], it is possible to visualize the attention weights to understand which facial regions the model is focusing on during the prediction process.

Interpretability of the model can also be enhanced by incorporating prior knowledge or physical context information into the model. For example, in facial soft tissue prediction after orthognathic surgery, the experience and knowledge of medical experts can be combined.

The improvements in data insufficiency, overfitting, and interpretability could hopefully provide us with more valuable planning tools to accurately and rapidly simulate the soft tissue appearance after orthognathic surgery, ultimately facilitating the widespread use of low-cost medical care and more precise surgery. With the help of DL, the ideal soft tissue position can be determined first, and then the amount and direction of bone movement required to achieve the soft tissue position can be calculated to provide suggestions for surgery. At present, there are more studies on soft tissues prediction concerning skeletal Class III than skeletal Class II, so future research on Class II patients should be strengthened. For individual patient prediction, FEM is currently recommended, yet for wide-range practice, training DL algorithms is recommended.

This narrative review, while providing a comprehensive overview of the existing literature on the prediction of soft tissue morphology after orthognathic surgery, is not without its limitations. Firstly, the scope of this review is confined to studies published in English. This deliberate focus, while ensuring a manageable dataset, inevitably excludes potentially valuable research published in other languages. Secondly, the data sources utilized in this review are limited to PubMed. This selection bias could lead to the exclusion of emerging research that may offer unique insights. Additionally, the narrative approach adopted in this review, as opposed to a systematic review or meta-analysis, introduces a degree of subjectivity. The absence of strict inclusion and exclusion criteria means that the selection and interpretation of studies are influenced by the reviewers' expertise and judgment. This subjectivity may result in the overemphasis of certain studies or the omission of others, potentially affecting the robustness of the conclusions drawn. Future research should strive to address these gaps by incorporating a broader range of studies, employing more rigorous review methods, and continuously updating the body of knowledge to reflect the latest findings.

## 5. Conclusions

The trend of soft tissue prediction after orthognathic surgery is from 2D to 3D prediction and from finite point prediction to point cloud simulation (visualization). Traditional 3D prediction methods have the main problems of low accuracy, slow speed, and high cost, hence hardly meet clinical needs. DL frameworks have shown broad prospects in improving the accuracy and speed of soft tissue simulation after orthognathic surgery, especially in lip region and severe deformities and complex surgery. DL prediction is mainly limited by data insufficiency, overfitting, and the lack of interpretability. Establishing cloud databases with shared resources globally to increase sample size and improve sample quality is the basis for improving DL prediction accuracy in key areas. Efforts should also be taken to deal with overfitting and low interpretability problems by improving model algorithm and optimizing the model performance. DL methods for facial point cloud prediction, perhaps supplemented by lip prediction with FEM, may be the most promising and practical method in the long run.

## Data Availability

Data sharing not applicable to this article as no datasets were generated or analyzed during the current study.
